# A Semiparametric Bivariate Probit Model for Joint Modeling of Outcomes in STEMI Patients

**DOI:** 10.1155/2014/240435

**Published:** 2014-04-01

**Authors:** Francesca Ieva, Giampiero Marra, Anna Maria Paganoni, Rosalba Radice

**Affiliations:** ^1^Department of Mathematics, Università Degli Studi di Milano, Via Saldini 50, 20133 Milano, Italy; ^2^Department of Statistical Science, University College London, Gower Street, London WC1E 6BT, UK; ^3^Modeling and Scientific Computing (MOX), Department of Mathematics, Politecnico di Milano, Via Bonardi 9, 20133 Milano, Italy; ^4^Department of Economics, Mathematics and Statistics, Birkbeck, University of London, Malet Street, London WC1E 7HX, UK

## Abstract

In this work we analyse the relationship among in-hospital mortality and a treatment effectiveness outcome in patients affected by ST-Elevation myocardial infarction. The main idea is to carry out a joint modeling of the two outcomes applying a Semiparametric Bivariate Probit Model to data arising from a clinical registry called STEMI Archive. A realistic quantification of the relationship between outcomes can be problematic for several reasons. First, latent factors associated with hospitals organization can affect the treatment efficacy and/or interact with patient's condition at admission time. Moreover, they can also directly influence the mortality outcome. Such factors can be hardly measurable. Thus, the use of classical estimation methods will clearly result in inconsistent or biased parameter estimates. Secondly, covariate-outcomes relationships can exhibit nonlinear patterns. Provided that proper statistical methods for model fitting in such framework are available, it is possible to employ a simultaneous estimation approach to account for unobservable confounders. Such a framework can also provide flexible covariate structures and model the whole conditional distribution of the response.

## 1. Introduction

Multiple outcomes are often used to properly characterize an effect of interest. Nevertheless, it often happens that the outcome of main interest is difficult or even impossible to measure. In general, realistic quantification of the effect of a predictor of interest on a particular response variable can be a difficult task in statistical analysis based on observational data. A solution is to control for confounders, that is, variables that are associated with both covariates and response. However, important confounders may be either unknown or too expensive to measure or not easily quantifiable (*unobservable confounders*). As pointed out in [1], this problem, which is known as* endogeneity* of the explanatory variable of interest, poses serious limitations to covariate adjustment since the use of classical techniques may yield biased and inconsistent estimates. Further issues which deserve attention are the possible presence of nonlinear covariate response relationships and how these change when considering the whole response variable distribution. There are many methods in the literature that can account for confounders. These include conditional approaches (e.g., stratification and model adjustment) and marginal approaches (e.g., matching and reweighting). For a review of these techniques see [2]. It should be noted, however, that these techniques do not account for unobserved confounding. Instrumental variable techniques are widely used for isolating the effect of a given predictor in the presence of unobserved confounding (see, among others, [3] and references therein). They are also increasingly used in epidemiological and medical studies [4]. The longest established Instrumental Variable (IV) estimators for binary outcome and treatment variables are the Generalized Method of Moment (GMM) [5], Maximum Likelihood (ML) [6], and Structural Mean Model (SMM) [7]. Among the ML estimators, the Recursive Bivariate (RB) probit model, introduced by [1], represents an effective way to estimate the effect that a binary regressor has on a binary outcome in the presence of unobservables. The semiparametric version of BP (SBP) is an important extension since undetected nonlinearity can have severe consequences on the estimation of covariate effects; see [8], where an example of a penalized maximum likelihood fitting procedure to estimate the recursive bivariate probit model with nonlinear confounder-response relationships is proposed.

The motivating problem of this work arises from the clinical context, where multiple outcomes are often used in order to characterize the patient's status or the performances of health care service with respect to patients' management. This is a framework where unobservable confounders are very popular as well.

In such a context, during the last decade, the increased capability of data collection has made available a huge amount of information about procedures and outcomes. More and more often multiple outcomes are measured in order to characterize treatment effectiveness or to evaluate the impact of large policy initiatives. The case study considered in the following concerns patients affected by ST-Elevation Myocardial Infarction (STEMI) and admitted to any hospital of Lombardia, the Italian regional district whose capital is Milan. Data come from a clinical registry named STEMI Archive [9, 10], which is a result of a wider comprehensive project (The Strategic Program “Exploitation, integration and study of current and future health databases in Lombardia for Acute Myocardial Infarction”).

In general, clinical registries and administrative databases are more and more often used nowadays to answer epidemiological enquiries (see [11–16], among others). The idea is to use information collected possibly with different purposes in order to analyze the efficacy and efficiency of the health care system on patients' outcomes. Thus, integrated health care systems for data collection measuring multiple outcomes play a fundamental role in complex clinical environments. Studies like those reported in [13–15] focus on data arising from REAL (Registro Angioplastiche dell'Emilia Romagna) and SCAAR (Swedish Coronary Angiography and Angioplasty Registry), respectively. These registries date back more than ten years and are consequently more structured and up-to-dated automatically with respect to STEMI Archive. Nevertheless, they are focused on the procedures (in particular, the Angioplasty and treatment of coronary in-stent restenosis), employed in the treatment of Acute Coronary Syndromes as a selection criterion for including patients in the study. Oppositely the main criterion to select eligible patients for STEMI Archive is the pathology diagnosis, being this registry more focused on imitating the classic epidemiological collections, though starting from observational data. Another difference between STEMI Archive and other registries lies in the collected information. In fact much organizational information related to hospital performances is gathered in it, in order to monitor the main process indicators in terms of time to intervention. Also concerning the goal of enhancing the integration of different sources of health information that is common to STEMI Archive, REAL, and SCAAR, STEMI Archive aims to do it also in order to automate and streamline clinicians' work flow, so that data collected once can be used multiple times for different aims. Specifically, they can serve for measuring performances of health care systems, for understanding how hospitals work, and for increasing efficacy of healthcare offer in terms of costs and patterns of care, for specific epidemiological enquires, and so on.

In STEMI Archive can be found. It consists of a clinical collection of data related to patients admitted in all hospitals of Regione Lombardia with STEMI diagnosis. As mentioned before one of the innovative contents of this survey is represented by process indicators recorded in it. They can be used to evaluate treatment times with the aim of designing a preferential therapeutic path to reperfusion in STEMI patients. In this sense, this survey represents an instrument both for epidemiological enquiries and for organizational optimization of the cardiological health care networks, quantifying the policies effects on multiple outcomes measured at patient's level. Within the data available from the STEMI Archive, there are two binary outcomes of interest for the present study: in-hospital mortality and reperfusion efficacy. The first one indicates if a patient is discharged alive from hospital. The second one indicates if a reduction greater than 70% of the ST-segment (The ECG signal can be divided into different waves and segments, delimited by some relevant points (landmarks), listed in alphabetical order starting from letter P. The *P* wave represents atrial depolarization; the ventricular depolarization causes the QRS complex that is followed by the ST segment. The ventricular depolarization is responsible of the *T* and the *U* waves. See [17], among others, for details.) elevation in the Electrocardiographic signal has been achieved after 1 hour from the reperfusion procedure, that is, the primary angioplasty (Percutaneous Transluminal Coronary Angioplasty or PTCA). These outcomes are clearly correlated and the interest lies in their joint modelling in order to accomplish a manyfold goal:

1. performance evaluation (in terms of in-hospital survival) of the health care structure the patients are admitted to;
2. quantification of the influence of procedural variables on outcomes: how does the management of the patterns of care affect the quality of life after discharge?
3. evaluation of the relationship between outcomes, that is, success in reperfusion practices and mortality, taking advantage of the joint modeling of their dependence on categorical and continuous covariates.

The paper is then organized as follows: in Section 2 we present the recursive bivariate probit model proposed by [8], highlighting the aspects that make such a model particularly suitable for carrying out the analysis of STEMI Archive data; in Section 3 the case-study is described and results of the analysis are proposed.  Section 4 contains the discussion of results and conclusions. All the analyses have been carried out using R software, version 3.0.2, and in particular we refer to the R-package SemiParBIVProbit, presented in [18].

## 2. Recursive Bivariate Probit Model

The bivariate probit model is a natural extension of probit regression model, where the disturbances of the two equations are assumed to be correlated in the same spirit as the seemingly unrelated regression model [6]. The recursive version of the bivariate probit allows us to estimate the effect of interest while accounting for unobserved confounders [1, 19]. The general specification is

(1) $$\begin{array}{c} y_{1i}^{*}=x_{1i}^{T}\alpha_{1}+\varepsilon_{1i},\\ y_{2i}^{*}=\gamma y_{1i}+x_{2i}^{T}\alpha_{2}+\varepsilon_{2i},\\ i=1,\ldots ,n, \end{array}$$

where *n* denotes the sample size and *y*_1i_* and *y*_2i_* are continuous latent variables (in the case of interest, the reperfusion efficacy score and propensity to die, resp.) which determine the observed binary outcomes *y*_1*i*_ (death or alive) and *y*_2*i*_ (procedure considered effective or not) through the rule *y*_*vi*_ = 1_{*y*_vi_*>0}_, for *v* = 1,2. Moreover, **x**_1*i*_^*T*^ = (1, **x**_12*i*_,…, **x**_1*P*_1_*i*_) is the *i*th row vector of the *n* × *P*_1_ model matrix **X**_1_, including variables related to the statistical units, in the following patients, like age, her/his total ischaemic time, killip class, and a measure of her/his ejection fraction at the entrance, and **α**_1_ is a parameter vector. Similarly, **x**_2*i*_^*T*^ is the *i*th row vectors of the *n* × *P*_2_ model matrix **X**_2_, **α**_2_ is a coefficient vector, and *γ* is the parameter of the endogenous binary variable *y*_1*i*_. The error terms (*ε*_1*i*_, *ε*_2*i*_) are assumed to follow the distribution *𝒩*([0,0], [1, *ρ*, *ρ*, 1]), where *ρ* is the correlation coefficient and the error variances are normalized to unity since the parameters in the model can only be identified up to a scale coefficient (e.g., [6]). To identify the parameters of the second equation in (1), it is typically assumed that the exclusion restriction on the exogenous variables holds. That is, the covariates in the first equation should contain at least one or more regressors (usually referred to as* instruments*) not included in the second equation. These regressors have to induce variation in *y*_1*i*_, have not to directly affect *y*_2*i*_, and have to be independent of (*ε*_1*i*_, *ε*_2*i*_) given covariates. However, as shown in [20, 21], the presence of this restriction may not be necessary to obtain consistent estimates of the model parameters.

Reference [8] proposed an extension of this model which allows for flexible functional dependence of the responses on continuous covariates: the semiparametric recursive bivariate probit model. This extension is important because the neglect of the presence of nonlinearity may have severe consequences on the estimation of covariate effects [22]. The semiparametric version of the classic bivariate probit can be written as

(2) y1i∗=x~1iTδ1+∑k1=1K1s1k1(x˘1k1i)+ε1i,y2i∗=γy1i+x~2iTδ2+∑k2=1K2s2k2(x˘2k2i)+ε2i,              i=1,…,n,

where ${\tilde{x}}_{1i}^{T}=\left(1,{\tilde{x}}_{12i},\ldots ,{\tilde{x}}_{1Q_{1}i}\right)$ is the *i*th row vector of ${\tilde{X}}_{1}$, the *n* × *Q*_1_ model matrix for the parametric model components (such as intercept, dummy, and categorical variables), with corresponding parameter vector **δ**_1_, and the *s*_1*k*_1__ are unknown smooth functions of the *K*_1_ continuous covariates ${\overset{˘}{x}}_{1k_{1}i}$, which, like age and total ischaemic time, could have a nonlinear influence on the corresponding outcome. Similarly, ${\overset{˘}{x}}_{2i}^{T}$ is the *i*th row vectors of the *n* × *Q*_2_ model matrix ${\overset{˘}{X}}_{2}$, with coefficient vector **δ**_2_, and the *s*_2*k*_2__ are unknown smooth terms of the *K*_2_ continuous regressors ${\overset{˘}{x}}_{2k_{2}i}$, in what follows, the measure of ejection fraction. Smooth terms are subject to identifiability constraints such as $\sum_{i}s_{vk_{v}}({\overset{˘}{x}}_{vk_{v}i})=0$, *v* = 1,2, *k*_*v*_ = 1,…, *K*_*v*_. The smooth functions are approximated using regression splines (e.g., [23]). Here, function $s_{k}({\overset{˘}{x}}_{ki})$, where subscript *v* has been suppressed to avoid clutter, is given by $\sum_{j=1}^{J_{k}}\beta_{kj}b_{kj}({\overset{˘}{x}}_{ki})=b_{k}{\left({\overset{˘}{x}}_{ki}\right)}^{T}\beta_{k}$, where the $b_{kj}({\overset{˘}{x}}_{ki})$ are known spline basis functions, with corresponding regression parameters *β*_*kj*_, *J*_*k*_ is the number of spline bases, $b_{k}({\overset{˘}{x}}_{ki})$ is a vector consisting of the basis functions evaluated at ${\overset{˘}{x}}_{ki}$, that is, $b_{k}{\left({\overset{˘}{x}}_{ki}\right)}^{T}=\left\{b_{k1}({\overset{˘}{x}}_{ki}),\ldots ,b_{kJ_{k}}({\overset{˘}{x}}_{ki})\right\}$, and **β**_*k*_ is the corresponding parameter vector. Basis functions are typically chosen to have convenient mathematical properties and good numerical stability. Possible choices include B-splines, cubic regression, and thin plate regression splines (see, e.g., [24] for an overview). Based on this representation, the equations in (2) can be written as $y_{1i}^{*}={\tilde{x}}_{1i}^{T}\delta_{1}+b_{1i}^{T}\beta_{1}+\varepsilon_{1i}=\eta_{1i}+\varepsilon_{1i}$, and $y_{2i}^{*}=\gamma y_{1i}+{\tilde{x}}_{2i}^{T}\delta_{2}+b_{2i}^{T}\beta_{2}+\varepsilon_{2i}=\eta_{2i}+\varepsilon_{2i}$, where, for *v* = 1,2, $b_{vi}^{T}=\left\{b_{v1}{\left({\overset{˘}{x}}_{v1i}\right)}^{T},\ldots ,b_{vK_{v}}{\left({\overset{˘}{x}}_{vK_{v}i}\right)}^{T}\right\}$, **β**_*v*_^*T*^ = (**β**_*v*1_^*T*^,…, **β**_*vK*_*v*__^*T*^) and *η*_*vi*_ has the obvious definition.

### 2.1. Estimation and Inference

In the bivariate probit model the data identify the four possible events (*y*_1*i*_ = 1, *y*_2*i*_ = 1), (*y*_1*i*_ = 1, *y*_2*i*_ = 0), (*y*_1*i*_ = 0, *y*_2*i*_ = 1), and (*y*_1*i*_ = 0, *y*_2*i*_ = 0) with probabilities *p*_11*i*_ = Φ_2_(*η*_1*i*_, *η*_2*i*_; *ρ*), *p*_10*i*_ = Φ(*η*_1*i*_) − *p*_11*i*_, *p*_01*i*_ = Φ(*η*_2*i*_) − *p*_11*i*_, and *p*_00*i*_ = 1 − *p*_11*i*_ − *p*_10*i*_ − *p*_01*i*_, where Φ and Φ_2_ are the distribution functions of a standardized univariate normal and a standardized bivariate normal with correlation *ρ*, respectively. Therefore, the log-likelihood function is

(3) ℓ(θ)=∑i=1n{y1iy2ilog⁡⁡(p11i)+y1i(1−y2i)log⁡⁡(p10i)+(1−y1i)y2ilog⁡⁡(p01i)+(1−y1i)(1−y2i)log⁡⁡(p00i)},

where ***θ*** ^*T*^ = (**δ**_1_^*T*^, **β**_1_^*T*^, *γ*, **δ**_1_^*T*^, **β**_2_^*T*^, *ρ*) according to the notation introduced in the previous section. When using regression splines, to avoid overfitting, the model parameters are typically estimated by maximization of

(4) $$\begin{array}{c} \ell_{p}\left(\theta \right)=\ell \left(\theta \right)-\frac{1}{2}\beta^{T}S_{\lambda }\beta , \end{array}$$

where **β** ^*T*^ = (**β**_1_^*T*^, **β**_2_^*T*^), **S**_***λ***_ = ∑_*v*=1_^2^∑_*k*_*v*_=1_^*K*_*v*_^ *λ*_*vk*_*v*__ **S**_*vk*_*v*__, and the **S**_*vk*_*v*__ are positive semidefinite known square matrices measuring the (second-order, here) roughness of the smooth terms in the model; that is, **β** ^*T*^(∑_*v*=1_^2^∑_*k*_*v*_=1_^*K*_*v*_^ *λ*_*vk*_*v*__ **S**_*vk*_*v*__), $\beta =\sum_{v=1}^{2}\sum_{k_{v}=1}^{K_{v}}\lambda_{vk_{v}}\int f_{vk_{v}}^{\prime \prime }{\left({\overset{˘}{x}}_{vk_{v}}\right)}^{2}d{\overset{˘}{x}}_{vk_{v}}$. The *λ*_*vk*_*v*__ are smoothing parameters controlling the trade-off between fit and smoothness. Given values for *λ*_*vk*_*v*__, maximization of (4) is straightforward. However, smoothing parameter estimation has to be settled in practice. This usually involves the use of specialized numerical routines minimizing, for instance, a prediction error criterion so that the estimated smooth functions are as close as possible to the true functions. In the current context, multiple smoothing parameter estimation is achieved by minimization of the approximate unbiased risk estimator [25]. Full computational details can be found in [8].

The inferential theory for models involving penalized regression splines is not standard. This is because of the presence of penalties which undermines the use of classic asymptotic results for practical modeling. Confidence intervals (CIs) for the components in the semiparametric bivariate probit model can be constructed using the well-known Bayesian “confidence” intervals typically employed in a generalized additive model context (e.g., [26, 27]). Interval calculations are therefore based on $\theta |y\dot{∽}𝒩(\hat{\theta },V_{\theta })$, where **y** contains the response vectors, $\hat{\theta }$ is the estimate of ***θ***, and **V**_***θ***_ represents the inverse of the penalized information matrix obtained at convergence of the algorithm. Given this result, CIs for linear and nonlinear functions of the model parameters can be easily obtained. Note that, for parametric model components, using the result above is equivalent to using classic likelihood results since such terms are not penalized. Also, there is no contradiction in fitting model (2) by penalized log-likelihood estimation and then constructing CIs adopting a Bayesian approach, and such a procedure has been employed many times in the literature (e.g., [23, 26]).

Note that in the absence of smooth functions in the model, as in (1), classic unpenalized maximum likelihood estimation can be reliably employed and traditional frequentist results used for inference. However, it is not possible to know whether smooth components are required before the analysis. In fact, using a more flexible model can help reducing the risk of misspecification due to undetected nonlinearity, which, as mentioned earlier, can have severe consequences on parameter estimation [22].

### 2.2. Average Treatment Effect

Since latent variables do not typically have well-defined units of measurements, parameter *γ* in model (1) may not be interpretable. For this reason the effect of the treatment *y*_1*i*_ on the response probability *P*(*y*_2*i*_ = 1 | *y*_1*i*_) is calculated. This can be done using the average treatment effect (ATE; e.g., [3]). Given estimates for the model components, the ATE can be estimated as follows:

(5) $$\begin{array}{c} \frac{1}{n}\sum_{i=1}^{n}\frac{\Phi_{2}\left({\hat{\eta }}_{2i}^{\left(y_{1i}=1\right)},{\hat{\eta }}_{1i};\hat{\rho }\right)}{\Phi \left({\hat{\eta }}_{1i}\right)}-\frac{\Phi_{2}\left({\hat{\eta }}_{2i}^{\left(y_{1i}=0\right)},-{\hat{\eta }}_{1i};-\hat{\rho }\right)}{1-\Phi \left({\hat{\eta }}_{1i}\right)}, \end{array}$$

where ${\hat{\eta }}_{2i}^{\left(y_{1i}=r\right)}$ indicates the linear predictor evaluated at *r* equal to 1 or 0. The interpretation of this measure is straightforward; it tells us how the probability of *y*_2*i*_ = 1 (of dying) changes if *y*_1*i*_ = 1 (the procedure the patient underwent was considered effective) as compared to *y*_1*i*_ = 1 (the procedure was not effective).

Coefficient *ρ* is also of interest as it is useful to ascertain the presence of unobserved confounding (endogeneity). Specifically, *ρ* can be interpreted as the correlation between the unobserved confounders in the two equations (e.g., [28]). If *ρ* = 0 then *ε*_1*i*_ and *ε*_2*i*_ are uncorrelated and hence there is no problem of endogeneity. In this case, estimation of the second equation in either (1) or (2) will yield consistent parameter estimates. Moreover, if the model (1) or (2) was fitted with intercepts only, it turns out that *ρ* is precisely the tetrachoric correlation, that is, a Pearson correlation between two bivariate normal variables that have been observed on a dichotomous scale [29]. CIs can be obtained using the delta method (see [30]).

## 3. Case Study

In this section we present the analyses carried out fitting a semiparametric bivariate probit model like in (2) to the data arising from STEMI Archive, the clinical registry gathering patients admitted with ST-segment Elevation Myocardial Infarction diagnosis in any hospital of Regione Lombardia district. A complete description of this clinical registry is provided in [10], where the Archive is presented together with the motivating clinical setting. Among the most important patient information provided by this clinical registry, there are as follows:

- *mode of admission*, that is, if a patient reaches the hospital on her/his own or delivered by three different types of rescue units of 118 (the national toll-free number for emergencies);
- *demographic features*, like age and sex;
- *clinical appearance*, that is, variables describing the patient's status at admission. Among others, we focus on killip class (binary variable categorizing the severity of infarction into 0 = less severe and 1 = more severe) and Ejection Fraction (EF);
- *risk factors*, like hypertension, diabetes, smoking, and chronic kidney disease (CKD);
- *times to treatment* (on/off hours),* times to treatment*,* times to intervention,* and all the* process indicators* concerned with pre- and in-hospital phase (Symptom Onset to Door time (OD), Door to Balloon time (DB), total ischaemic time (OB), etc.);
- *clinical outcomes*, that is, in-hospital mortality and treatment efficacy (STres), quantified by a reduction of ST segment elevation in the ECG.

We focus our readings on patients who underwent primary transluminal coronary angioplasty (PTCA), the most common reperfusion procedure for acute myocardial infarctions. The population considered for the following analyses consists of 1069 statistical units.

In this application, the binary outcomes of interest are patients' in-hospital mortality and the efficacy of the reperfusion treatment they undergo. The efficacy is determined by the reduction of ST segment elevation one hour after the surgery: if the reduction is over 70% the procedure is considered effective. Thus it is clear why the joint modelling of in-hospital mortality and reperfusion efficacy makes sense. Not only are they likely to be correlated, but also a strong clinical interest lies in quantifying the degree of correlation among these two. Moreover, reperfusion efficacy indicator is a binary variable whose values depend on the latent recovered ability of the coronary arteries to work properly. So the framework presented in [8] seems to be the proper way to address the problem of interest.

The variable selection has been carried out according to both clinical knowhow and the statistical stepwise approach, similar to what is proposed in [31–34]. Then the model (2) has been fitted to STEMI Archive data with the following specifications:

1. for the outcome **y**_1_* (the* reperfusion efficacy*, STres) we retained a binary variable (${\tilde{x}}_{11}$) indicating if the patient reaches the hospital on her/his own (access) and two continuous variables (${\overset{˘}{x}}_{11}$ and ${\overset{˘}{x}}_{12}$), being the age (age) of the patient and her/his total ischaemic time (O2B, i.e., the time between the symptom onset and the PTCA procedure), respectively;
2. for the outcome **y**_2_* (the* in-hospital mortality*, mortality) we retained a categorical variable (${\tilde{x}}_{21}$) indicating the patient's killip class (killip) and a continuous variable (${\overset{˘}{x}}_{21}$) measuring her/his ejection fraction (EF) at the entrance.

Therefore, for *i* = 1,…, 1069, model (2) becomes:

(6a) STres1i∗=δ10+δ11×accessi+δ12×agei+s11(02B1i)+ε1i,

(6b) Mortality1i∗=δ20+δ21×STres+δ22×killip+δ23×EF+ε2i.

Table 1 shows the estimates provided by the bivariate probit model for the mortality outcome ((6b), Table 1(b)) and the indicator of successful reperfusion therapy ((6a), Table 1(a)).

It can be noticed that all the selected covariates are significant. In particular, the treatment efficacy decreases as the age increases, as expected. The way of admission of patients delivered by 118 eases the good prognosis, too. It is worth noting that the total ischaemic time effect is nonlinear, being the smoother degrees of freedom significantly greater than one. This confirms the clinical knowhow according to which the way the delay affects the treatment efficacy is definitely nonlinear [35].

Concerning the mortality outcome, as expected, the more severe the infarction (quantified by killip class), the higher the mortality. Also a reduced ejection fraction plays the role of increasing the mortality, as it is known by clinical practice.

The estimated correlation coefficient of the recursive bivariate probit model is equal to 0.394 and it is significantly different from zero at the 5% level (CI(*ρ*) = (0.0637,0.644)), hence supporting the presence of unobserved confounders. In fact, it is usual that a lot of unexplained variability exists in complex healthcare processes where patterns of care consist of multiple phases. It derives from the variability existing at patient's level plus a variability induced by the complex process of patient's management. Then it is crucial to find correlations and to identify which procedures clinicians can act upon in order to improve the process of care.

Table 2 shows ATE estimates obtained using SBP. For completeness we also report the unadjusted estimate and that obtained using an additive probit model (AP). AP is a model which accounts for observed confounders but not unobserved confounders. Under this setting ATE has been estimated by fitting the equation of interest alone whereas the unadjusted estimate does not account for both observed and unobserved confounders.

It can be observed that all point estimates are negative which is consistent with the reasoning that the better the efficacy, the lower the mortality probability. However, the unadjusted ATE effect is significantly greater than those obtained using SBP and AP. This difference is due to the fact that the former estimate does not adjust for both observed and unobserved confounders and hence should be regarded with suspicion. If we account for observed confounders only, then the effect becomes strongly nonsignificant whereas the CI of ATE changes considerably if we take into account the unobservables. The modification of the CI seems to suggest that with a greater number of observations, our tenet on ATE could be confirmed. Anyway, in this case results suggest that the presence of unobserved confounders detected by the bivariate model may be regarded as variables which do not interfere with the relationship of interest but whose presence inflates the variance of the estimates.

In this study we were concerned with the possible detrimental effect of unobserved confounders on the effect of interest (reperfusion efficacy on mortality). Based on our analysis, this does not seem to be an issue as the SBP and naive models produced similar point estimates. However, the use of a bivariate probit model may still be preferred as it may allow for more reliable inferences.

## 4. Discussion

It is more and more frequent in clinical practice that multiple outcomes are measured for properly characterizing an effect of interest in terms of diseases or for assessing health care policies and performances. Nevertheless, it often happens that (some) outcomes are difficult (even impossible) to be measured or that confounders are difficult to be accounted for when modeling such outcomes by means of suitable covariates. Instrumental variables are nowadays an established method for isolating the effect of a given predictor in the presence of unobserved confounding.

In this work we showed an application of a semiparametric bivariate probit model to a couple of binary outcomes representing the in-hospital mortality and an indicator of the reperfusion efficacy in patients affected by acute myocardial infarction. The efficacy is determined by the reduction of ST segment elevation one hour after the surgery: if the reduction is over 70% the procedure is considered effective. Data come from a clinical registry called STEMI Archive [9]. This case study claims for the joint modelling of the in-hospital mortality and reperfusion efficacy outcomes. It makes sense not only because they are likely to be correlated, but also because a strong clinical interest lies in quantifying the degree of correlation among these two. Moreover, reperfusion efficacy indicator is a binary variable whose values depend on the latent recovered ability of the coronary arteries to work properly. In this sense, this modelling strategy represents a step forward with respect to the results pointed out in [31] and then in [32, 36], since a joint modelling of correlated outcomes is possible, as well as parametric and nonparametric definition of the relationship between outcomes and covariates.

In fact there are many methods that can account for confounders, but many of these don not account for confounders. This approach's advantage over methods like Generalized Method of Moment (GMM) and Structural Mean Models (SMM) is twofold. First, semiparametric bivariate probit model allows for flexible functional dependence of the response variables on continuous covariates via the use of penalized regression splines. Unlike the classic parametric approach typically employed in these kinds of studies, a semiparametric specification allows us to flexibly model the effect of continuous covariates (e.g., the age of individuals) without making a priori assumptions (e.g., linearity or nonlinearity specified using quadratic or cubic polynomials). This reduces the risk of model misspecification due to undetected nonlinearity, which can have severe consequences on parameter estimation. Second, provided that the model assumptions are met, identification of the treatment effect is theoretically achieved even if an instrument is not included in the model [20, 21]. This paper does not employ those IV techniques for a variety of reasons. First, GMM and SMM do not perform well if they do not have a valid instrumental variable. More importantly, these procedures are not implemented in software-accessible code making it difficult for practitioners to use and interpret the results.

Results are strongly coherent with clinical practice. This enables a better comprehension of the disease-recovery dynamics and enables better predictions for new patients entering the study. In general, in complex processes such clinical ones where many sources of latent interactions may arise, accounting and adjusting for confounders is extremely important. This appears clearly looking at results reported in Table 2.

In general, diagnosis and management of AMI patients are difficult and may strongly benefit of the aid of statistical models that provide effective risk stratification of patients. In fact, flexible models that are able to properly profile patients adjusting for case mix and confounders are extremely of interest in the context of modern clinical practice, since the more accurate predictions and more reliable prognoses that they provide enable gaining insights of the economic burden of AMI, supporting an effective clinical decision making.

## Figures and Tables

**Table:** (a) Equation (6a)

	Coefficient	Estimate	Std. err.	*P* val.
i ntercept	*δ*_10_	1.3811	0.2488	<0.0000
access	*δ*_11_	0.2129	0.0933	0.0225
age	*δ*_12_	−0.0088	0.0036	0.0140

	Smooth term	Edf	Est. rank	*P* val.

*s*(O2B)	*s*_11_	1.356	2	0.0041

**Table:** (b) Equation (6b)

	Coefficient	Estimate	Std. err.	*P* val.
intercept	*δ*_20_	1.7708	0.4656	0.0001
STres	*δ*_21_	−1.2480	0.2109	<0.0000
killip	*δ*_22_	0.7223	0.2544	0.0045
EF	*δ*_23_	−0.0748	0.0119	<0.0000

**Table 2:** ATE estimates obtained by fitting the semiparametric bivariate probit and the naive approach, respectively.

ATE of	Estimate	CI
SBP	−2.05	(−4.88, 0.77)
AP	−1.92	(−20.63, 16.78)
Unadjusted	−4.46	(−6.32, −2.61)
